# Moderating Effect of Social Support Between Loneliness and Depression: Young-Old and Old-Old Adults

**DOI:** 10.1093/geroni/igab046.1138

**Published:** 2021-12-17

**Authors:** Sunghwan Cho, Kyuhyung Chung

**Affiliations:** 1 Virginia Commonwealth University, Richmond, Virginia, United States; 2 Semyung University, Semyung University, Ch'ungch'ong-bukto, Republic of Korea

## Abstract

Depression increases suicidal risk lowers quality of life in older adults. However, it is unknown how loneliness and depression are associated with young-old and old-older adults. This study examined association of loneliness and depression from the National Social Life, Health, and Aging Project (NSHAP) (2015-2016), estimating moderating effects of social support. The sample of this study was community dwelling Medicare beneficiaries aged 65+ (n=1,532): young-older adults (n=903) and old-older adults (n=629). Loneliness was measured by the Revised University of California, Los Angeles Loneliness Scale short form (3 items; young, M=.86, SD=.73; old, M=.87, SD=.67; range 0-3). Social support consists of two variables each measured by 4 items, spouse/partner support (young, M=2.29, SD=.50; old, M=2.26, SD=.51; range 0-3) and family support (young-old, M=2.19, SD=.52; old-old, M=2.23, SD=.52; range 0-3). Depression was measured by Center for Epidemiological Studies Depression scale (11 items, young, M=1.41, SD=.42; old, M=1.45, SD=.42; range 1-4). Multiple linear regression was used in this study, including relevant covariates. Findings indicated loneliness in both groups (young, p<.001; old, p<.001), spouse support in both groups (young, p<.001; old, p<.001) had statistical significance in depression. Family support in young-older adults (p<.05) had a statistical significance for depression. Interaction of loneliness and spouse support moderated the relationship between loneliness and depression in old-older adults (p<.05). Findings suggest old-older adults’ loneliness and depression could be soothed by spousal support. Spousal support could be important in that the informal caregiver is likely to focus on older adults with fragility at the end of their lives.

